# Renal oncometabolite L-2-hydroxyglutarate imposes a block in kidney tubulogenesis: Evidence for an epigenetic basis for the L-2HG-induced impairment of differentiation

**DOI:** 10.3389/fendo.2022.932286

**Published:** 2022-09-05

**Authors:** Mary Taub, Nader H. Mahmoudzadeh, Jason M. Tennessen, Sunil Sudarshan

**Affiliations:** ^1^ Biochemistry Department, School of Medicine and Biomedical Sciences, State University of New York at Buffalo, Buffalo, NY, United States; ^2^ Department of Biology, Indiana University, Bloomington, IN, United States; ^3^ Department of Urology, University of Alabama at Birmingham, Birmingham, AL, United States

**Keywords:** L-2-Hydroxyglutarate, proximal tubule, matrigel (MA), differentiation, renal cell carcinoma

## Abstract

2-Hydroxyglutarate (2HG) overproducing tumors arise in a number of tissues, including the kidney. The tumorigenesis resulting from overproduced 2HG has been attributed to the ability of 2HG alter gene expression by inhibiting α-ketoglutarate (αKG)-dependent dioxygenases, including Ten-eleven-Translocation (TET) enzymes. Genes that regulate cellular differentiation are reportedly repressed, blocking differentiation of mesenchymal cells into myocytes, and adipocytes. In this report, the expression of the enzyme responsible for L2HG degradation, L-2HG dehydrogenase (L2HGDH), is knocked down, using lentiviral shRNA, as well as siRNA, in primary cultures of normal Renal Proximal Tubule (RPT) cells. The knockdown (KD) results in increased L-2HG levels, decreased demethylation of 5mC in genomic DNA, and increased methylation of H3 Histones. Consequences include reduced tubulogenesis by RPT cells in matrigel, and reduced expression of molecular markers of differentiation, including membrane transporters as well as HNF1α and HNF1β, which regulate their transcription. These results are consistent with the hypothesis that oncometabolite 2HG blocks RPT differentiation by altering the methylation status of chromatin in a manner that impedes the transcriptional events required for normal differentiation. Presumably, similar alterations are responsible for promoting the expansion of renal cancer stem-cells, increasing their propensity for malignant transformation.

## 1 Introduction

Specific cancer cells including gliomas, secondary glioblastomas, and acute myeloid leukemia (AML) overproduce D-2-Hydroxyglutarate (D-2HG) due to point mutations in cytosolic Isocitrate Dehydrogenase 1 (IDH1) (1). Renal carcinomas similarly have been observed to overproduce 2HG; however, the L isoform is overproduced in these tumors, presumably due to reduced expression of L-2HG dehydrogenase, which normally oxidizes L-2HG to αKetoglutarate (αKG) (2). While a number of mechanisms have been proposed, the most validated mechanisms by which 2HG accelerates oncogenesis are epigenetic. 2HG potently inhibits αKG-dependent dioxygenases by preventing αKG binding (3). Included amongst the αKG-dependent dioxygenases which are affected are the Ten-Eleven Translocation (TET) enzymes, which demethylate 5-methylcytosine (5mC) residues in genomic DNA (1), as well as the Jumonji C (JmjC) domain-containing histone demethylases (JMDHs). The consequence of TET inhibition is hypermethylation of DNA, while the consequence of JMDH inhibition is hypermethylation of histones. Ultimately, the increased DNA and histone methylation changes gene expression. Of particular interest in these regards is that these changes in gene expression have been observed to block cellular differentiation into adipocytes and muscle cells (4, 5). These observations have been made with cells that overproduce D-2HG due to IDH1mutations (1). However, the question has not been addressed as to whether L2HG-producing cells have a similar block in differentiation. This question is of particular importance with regard to clear cell renal carcinomas (CCRCCs), given that the studies of Shelar et al. (2) indicate that L2HG contributes to the development of these tumors.

Here we examine the hypothesis that elevated 2HG affects the differentiation of normal renal cells, altering their ability to undergo tubulogenesis. Of particular interest in these regards are renal proximal tubule (RPT) cells, the cells of origin of ccRCCs (6). In our previous studies, we observed that normal RPT cells, which have just been removed from the animal, form monolayer cultures that exhibit differentiated functions when cultured on plastic in defined medium (7). However, when RPT cells are cultured in a reconstituted basement membrane, matrigel, tubulogenesis occurs in response to either EGF or TGFα (8). Furthermore, the newly formed tubules possess the capacity for transepithelial transport (9), similar to developing nephrons (8, 10). Included amongst the initial events which occur during kidney development is the induction of the metanephric mesenchyme by WNT signals, followed by the induction of transporters specific to the RPT (11, 12). Notably, these developmental events are dependent upon DNA and histone methylation (13–16). For this reason, we have examined whether a) renal proximal tubulogenesis, and the expression of developmentally regulated transporter genes is altered by 2HG, and b) whether 2HG-mediated alterations can be attributed to changes in DNA and histone methylation.

## 2 Materials and methods

### 2.1 Materials

Dulbecco’s Modified Eagle’s Medium (DMEM), Ham’s F12 Medium (F12), growth factor depleted matrigel, soybean trypsin inhibitor, 0.05% EDTA/0.53 mM trypsin in phosphate-buffered saline (PBS), EGF, lipofectamine, siRNA, RNA-4PCR kits, TURBO DNase I, Superscript Vilo kits, DNA oligos, and tissue culture plasticware were from Thermo Fisher (Waltham, MA). TransIT-LT1 transfection reagent was from Mirus Biotechnology Co. (Madison, WI). Tissue culture plate inserts, 24 wells, with PET membranes, 8.0 µm, were from VWR (Radnor, PA). Monarch Genomic DNA Purification Kits were obtained from New England BioLabs (Ipswich, MA). Hybond-N+ membranes were from GE Healthcare Biosciences (Chicago, IL). Nitrocellulose membranes were from Bio-Rad Laboratories (Hercules, CA). The Sirius Western Bright detection kit was from Advansta, San Jose, CA. The 5-hydroxy methyl cytosine (5-hmC) rabbit polyclonal antibody was from Active Motif (Carlsbad, CA), and the rabbit polyclonal antibodies against di- and tri-methylated histones (included in Histone Sampler Kits 9783 and 9847) were from Cellular Signaling Technologies, Danver, MA. The rabbit anti-NPT2a antibody was from alpha Diagnostics International (San Antonio, TX), while the rabbit anti-L2HGDH antibody (Cat # 15707-1-AP) and the rabbit anti- SGLT2 antibody (Cat. # 24654-1-AP) were from Proteintech (Rosemont, IL). The mouse monoclonal anti-beta actin antibody was from Santa Cruz Biotech (Dallas, TX). The secondary antibodies (Horseradish Peroxidase (HRP)-coupled Goat anti-Rabbit and HRP-coupled Goat anti-Mouse), as well as the SsoAdvanced Universal Syber Green Supermix, were obtained from Bio-Rad Laboratories (Hercules, CA). Collagenase Class IV was from Worthington (Freehold, NJ). Western Blocking Reagent, produced by Roche, as well as bovine insulin, human transferrin, hydrocortisone, and other chemicals was from Sigma Aldrich Chemical Corp. (St. Louis, MO). Selenium was from Difco laboratories (Detroit, MI). New Zealand White rabbits, 4-5 lb, male, were from Charles River (Wilmington, MA). Prism 9 software was from GraphPad, Inc. (San Diego, CA).

### 2.2 Plasmids

The pLK0.1 TRC cloning vector (17), the pLKO.1-TRC vector (17), and the scramble shRNA vector in pLKO.1 (18) were obtained from Addgene (Watertown, Mass). The pMD2.G vector [expressing VSV-G envelop; Addgene plasmid # 12259; http://n2t.net/addgene:12259;RRID:Addgene_12259), and psPAX2 (a lentiviral packaging vector; Addgene plasmid # 12260; http://n2t.net/addgene:12260;RRID:Addgene_12260)] were gifts from Didier Trono.

### 2.3 Cell culture

The basal medium, which consists of a 50:50 mixture of Dulbecco`s Modified Eagle`s Medium and Ham’s F12 Medium containing 15 mM HEPES and 20 mM sodium bicarbonate (DMEM/F12) (pH 7.4), is supplemented with 5 µg/ml bovine insulin, 5 µg/ml human transferrin, 5 × 10^-8^ M hydrocortisone, 92 U/ml penicillin, and 0.01% kanamycin (i.e., Medium RK-1). Water used for medium and growth factor preparations was purified using a Milli-Q deionization system. Cultures were maintained in a humidified 5% CO_2_/95% air mixture at 37°C.

Primary rabbit RPT cell cultures were initiated from rabbit kidneys, as previously described (7). Animal use was reviewed and approved by the Institutional Animal Care and Use Committee of the State University of New York at Buffalo. After their removal from the animal, rabbit kidneys were perfused *via* the renal artery, with DMEM/F12 containing 0.5% iron oxide (w/v), until the kidney turned gray black in color. Renal cortical slices were removed, disrupted with a sterile glass homogenizer, and the material was separated sequentially through 253 µm and 83 µm nylon sieves. Tubules and glomeruli on the 83 µm sieve were transferred into DMEM/F12, glomeruli (containing iron oxide) removed with a stir bar, and remaining proximal tubules incubated in DMEM/F12 containing 0.05mg/ml collagenase IV/0.5 mg/ml soybean trypsin inhibitor (2′; 23°C). Dissociated tubules were centrifuged, resuspended in DMEM/F12, and plated into culture dishes (or 12-well plates) containing Medium RK-1. The medium was changed the day after plating, and every 2 days thereafter.

### 2.4 Treatment of primary cultures with either L2HGDH siRNA or L2HGDH shRNA

The sequence of rabbit L2HGDH stealth siRNA (UUACAGUACUCAUACAUGAGGGCUG, positive strand) and scrambled (scr) control stealth siRNA (UUAGGCAUGAACUCACAUGAGUCUG, positive strand) was determined using Stealth siRNA software (Thermo Fisher), whereas the sequence of rabbit L2HGDH Silencer Select siRNA (GAUGCUUACUGUUUUGGAAtt) was determined using Silencer Select siRNA software (Thermo Fisher). Silencer Select Negative Control siRNA #1 was used in parallel with L2HGDH silencer select siRNA. Primary RPT cells were transfected with either Rabbit L2HGDH siRNA or a Control siRNA (scrambled stealth siRNA) using lipofectamine, while transfections with L2HGDH Silencer Select siRNA or a Control siRNA (Silencer Select Negative Control siRNA #1, ThermoFisher) were conducted using lipofectamine RNAiMAX. Two days later, the cultures were either used experimentally or transfected a second time with the siRNAs.

A rabbit L2HGDH shRNA oligo, generated by RNAi Consortium Software, the Broad Institute, (CCGGAAGATGGGATGAAATATCCAATTCTCGAGTTGGATATTTCATCCCATCTTTTTTTG), was inserted into a pLK0.1 TRC cloning vector. The sequence was verified by the Roswell Park Cancer Institute Sequencing Facility (Buffalo, NY). The pLKO.1-TRC vector and the scramble shRNA vector in pLKO.1 were used controls. To prepare lentivirus, 292 T cells were cotransfected, using a TransIT-LT1 transfection agent, with a pLK0.1 TRC vector, pMD2.G, and psPAX2 followed by medium change (after 24 h). Medium containing virus was collected 48 and 72 h after transfection. and the virus was titered using HT1080. Primary RPT cell cultures were transduced with lentiviral particles, and transformants selected for 7 days using puromycin.

### 2.5 Matrigel cultures

Growth factor depleted matrigel, prepared as described by Taub et al. (8), was stored at -20°C. Prior to its use, matrigel was thawed and maintained at 4°C. Prior to the addition of the cultures, 12-well plates were coated with matrigel. Subsequently, monolayer cultures of primary RPT cell cultures were detached from their dishes using EDTA/trypsin. Trypsin action was inhibited using 0.1% soybean trypsin inhibitor in PBS. The cells were suspended in DMEM/F12 and pelleted at 500×*g* for 5 min. After resuspension in DMEM/F12, the cell number was determined using a Coulter counter, and the cells were added to matrigel at 4°C. The cells in matrigel were plated into individual wells of matrigel-coated 12 well plates at 2 × 10^4^ cells/well. The matrigel cultures were maintained in a humidified 5% CO_2_ incubator in a humidified 5% CO_2_/95% air environment at 37°C. DMEM/F12 medium containing 5 µg/ml bovine insulin, 5 µg/ml human transferrin (DMEM/F12-IT), and other pertinent factors (including 5 ng/ml EGF) was added the day after plating. The matrigel cultures were incubated with EGF, and/or other appropriate supplements. One week later, the number of tubules was determined in each of 25 microscope fields/well, in three wells per condition, and compared to control values in the absence of added growth factor (unless otherwise stated).

### 2.6 Realtime PCR

RNA was purified from the cultures using an RNA-4PCR kit. Subsequently, genomic DNA was removed using TURBO DNase I, and cDNA was synthesized using a Superscript Vilo kit. Transcripts were amplified using a BioRad CFX96 RealTime System using SsoAdvanced Universal Syber Green Supermix containing 5 µM forward and reverse primers complementary to cDNA templates. Ct values (obtained using BioRad software) were determined in quadruplicate. Relative mRNA levels were calculated using the Ct values, as described by Pfaffl (19) using beta actin mRNA as an internal control. Primers were designed by using Primer-BLAST (NCBI website) and synthesized by ThermoFisher.

### 2.7 Western analysis

Cell lysates were prepared in RIPA buffer containing protease inhibitors, as previously described (20), and protein levels were determined using the micro BCA protein assay (21). The samples, equalized with respect to protein, were separated by electrophoresis through 7.5% SDS/polyacrylamide gels, and transferred to nitrocellulose, as previously described (22). The blots were incubated first with a primary antibody and, subsequently, with an HRP-conjugated secondary antibody, also as previously described (22). Following an incubation with WesternBright Sirius Chemiluminescent HRP substrate, bands were visualized in a BioRad Chemidoc MP. Band intensities were compared using ImageLab Software.

### 2.8 5-Hydroxymethylcytosine slot blots

Slot blots were employed to probe for 5-hydroxymethylcytosine (5hmC) in genomic DNA, using serial dilutions of known quantities of genomic DNA, a semi-quantitative method described by Liu et al. (23) and Jia et al. (24). To summarize, genomic DNA was purified using a Monarch Genomic DNA Purification Kit and quantitated using a Nanodrop. In order to expose the bases, the DNA was denatured at 99°C for 5 min, and quick cooled. Dilutions of the samples were applied onto an Hybond-N+ membrane, using a HybriSlot Manifold, and baked at 80°C for 1 h. The membrane was blocked 1 h in 1% Blocking Solution (1% Western Blocking Reagent in Tris Buffered Saline (TBS)), followed by 1 h incubation with 5-hydroxy methyl cytosine (5-hmC) rabbit polyclonal antibody in 0.5% Blocking Solution. The membrane was washed twice in TBS + 0.1% Tween 20 (TBST) and incubated for 1 h in 0.5% blocking solution containing a Horseradish Peroxidase (HRP)-coupled Goat anti-Rabbit secondary antibody. After four washes with TBST, bands were developed using a Sirius Western Bright detection kit and visualized using a BioRad Chemidoc MP.

Following 5hmC blotting, the blots were stained with methylene blue, in order to visualize total DNA on the blots, within the limits of its sensitivity.

### 2.9 Analysis of histones

Primary RPT cell cultures in 60 mm dishes were treated with either a) L2HGDH stealth siRNA in parallel with scrambled (scr) control siRNA or b) L2HGDH Silencer Select siRNA in parallel with Silencer Select Negative Control #1 siRNA. Two days later, histones were extracted from the cultures using a Histone Extraction Kit (ab113476; Abcam, Cambridge, MA), and protein was determined as described by Scopes (25), employing a Nanodrop. Purified histones, equalized with respect to protein, were separated on 12.5% SDS-polyacrylamide gels, transferred to Nitrocellulose, and subjected to Western analysis (using rabbit polyclonal antibodies against di- and tri-methylated histones), as previously described (22).

### 2.10 Determination of L- and D-2HG, and glutamine

Primary RPT cell cultures in 100 mm dishes were treated with either a) L2HGDH Stealth siRNA or scrambled control siRNA, or b) L2GDH Silencer Select siRNA or Negative Control 1 siRNA, as described above. Two days later, the cultures were harvested as follows. The medium was changed 2 h prior to harvesting. The cultures were treated with EDTA/trypsin. Dislodged cells were transferred into a 2 ml screwtop tube (removing a sample to determine cell number). The cells were centrifuged (3,000×*g*), washed with PBS, and flash-frozen in dry ice/ethanol. Samples were then utilized for the determination of L- and D- 2HG levels. Subsequently, samples were homogenized in a bead mill homogenizer, L- and D-2HG were derivatized using acidified R-2-butanol, and derivatized L- and D-2HG were separated by GC-MS, as described by Li and Tennessen (26). The identity of the peaks for L- and D-2HG was verified using *L,D*-[2,3,3-^2^H_3_]-2-hydroxyglutarate) as an internal standard, and peak areas for L- and D-2HG were determined, also as described by Li and Tennessen (26), The final concentration of L- and D-2HG (determined in nanomoles) was standardized with respect to cell number, as determined using a Coulter Counter. Final values are averages of triplicate determinations +/- the SEM.

In order to examine the effect of glutaminase inhibitor CB-839 on glutamine levels, intracellular glutamine and glutamate were determined in triplicate cultures using the Promega Glutamine/Glutamate-Glo Assay. Cultures were grown in 96-well plates in Medium RK-1. The medium was changed to either Medium RK-1 with diluent (DMSO), or Medium RK-1 supplemented with 1 µM CB-839. After a 3-day incubation, the cultures were lysed in 0.1N HCl. A portion of the lysate was treated with glutaminase, while the other portion was untreated. Subsequently, the glutamate level was determined both in glutaminase-treated and untreated lysates using the Glutamate-Glo Assay. Emitted light was quantitated using a Biotek Plate Reader. The glutamine level was determined by subtracting the glutamate level measured in an untreated lysate from the glutamate level determined in the portion of the lysate treated with glutaminase. Values are averages +/- SEM of triplicate determinations.

### 2.11 Transwell migration assay

Primary RPT cells were trypsinized and plated (5 × 10^4^ cells/0.5 ml) into matrigel coated tissue culture plate inserts with PET membranes in 12-well plates. DMEM/F12 containing chemoattractant was added to the bottom chamber. The next day, the transwell was removed and washed with PBS, and the cells were removed from the membrane side facing the upper chamber using a cotton swab. After fixing the cells with formalin, the transwell was washed twice with PBS, the cells were stained with Hoechst (10 µg/ml), and the transwells were washed twice with PBS. Images were captured (in at least 25 microscope fields) using a Zeiss Axio Observer Inverted Microscope. The cells in each of the images were automatically counted using NIH ImageJ software. The average number of cells/10 microscope fields was determined in each of the three transwells/condition.

### 2.12 Statistical analysis

Statistical analyses were conducted using Prism software. Statistical results are expressed as means +/- SEM. Statistical differences between groups were determined using a two-tailed t-test. Differences between means were considered statistically significant when p < 0.05.

## 3 Results

### 3.1 Effect of L2HGDH KD on tubulogenesis

**Figure 1:**
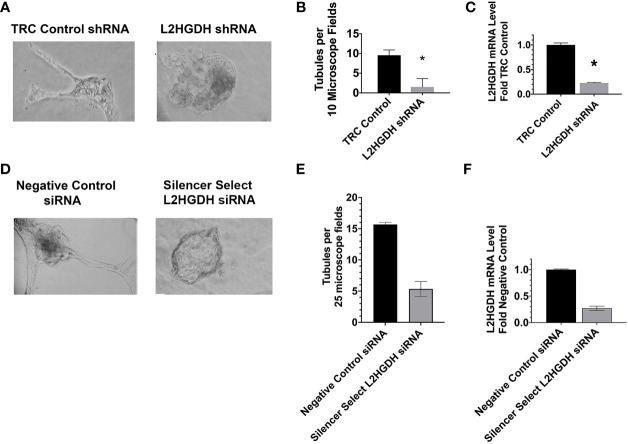
Inhibition of tubulogenesis by L2HGDH shRNA AND L2HGDH siRNA. Primary RPT cell cultures were either **(A)** transduced with lentivirus containing either L2HGDH shRNA or Control TRC shRNA vectors, or **(D)** transfected with either Silencer Select L2HGDH siRNA, or Negative Control siRNA. Prior to culturing in matrigel, the shRNA transduced cells were selected 1 week with 1.6 µg/ml puromycin, while the siRNA transfected cells were cultured 1 day to allow for gene expression. Subsequently, the primary cultures were trypsinized and passaged into matrigel in DMEM/F12-IT further supplemented with 5 ng/ml EGF, as described in Materials and Methods. Representative microscope fields of matrigel cultures are illustrated including (A1) matrigel cultures transduced with either Control TRC shRNA or L2HGDH shRNA, and (B1) matrigel cultures transfected with either Silencer Select L2HGDH siRNA or Negative Control siRNA. **(B)** the effect of the L2HGDH shRNA on number of tubules was quantitated, relative to Control shRNA, and **(C)** the relative levels of L2HGDH mRNA determined in the two conditions. Similarly, in **(E)** the effect of Silencer Select L2HGDH siRNA on the number of tubules was quantitated, relative to Negative Control siRNA, and **(F)** the relative levels of L2HGDH mRNA determined in the same 2 conditions. Values are averages +/- SEM of triplicate determinations. (*) p < 0.05 relative to either the TRC control shRNA **(B, C)** or the Negative Control siRNA **(E, F)**, respectively. Scale Bars, 50 µm.

The effect of an L2HGDH Knockdown (KD) on renal proximal tubulogenesis *in vitro* was examined. Towards these ends, primary RPT cell cultures were transduced with lentiviral particles containing vectors expressing either L2HGDH shRNA, or the Control TRC shRNA vector, and selected with puromycin. Subsequently, transduced cells were introduced into matrigel and cultured in the presence of EGF. **Figure 1A** shows tubules observed in Control TRC transduced cultures, as compared with the structures in cultures transduced with L2HGDH shRNA. Quantitative studies of parallel cultures indicated that both the number of tubules and the L2HGDH mRNA levels were reduced by 80% (**Figures 1B** **, C**, respectively). Similar results were obtained when primary RPT cell cultures were transfected with Silencer Select L2HGDH siRNA, in comparison with Negative Control siRNA (as shown in **Figures 1D** **–F**).

**Figure 2:**
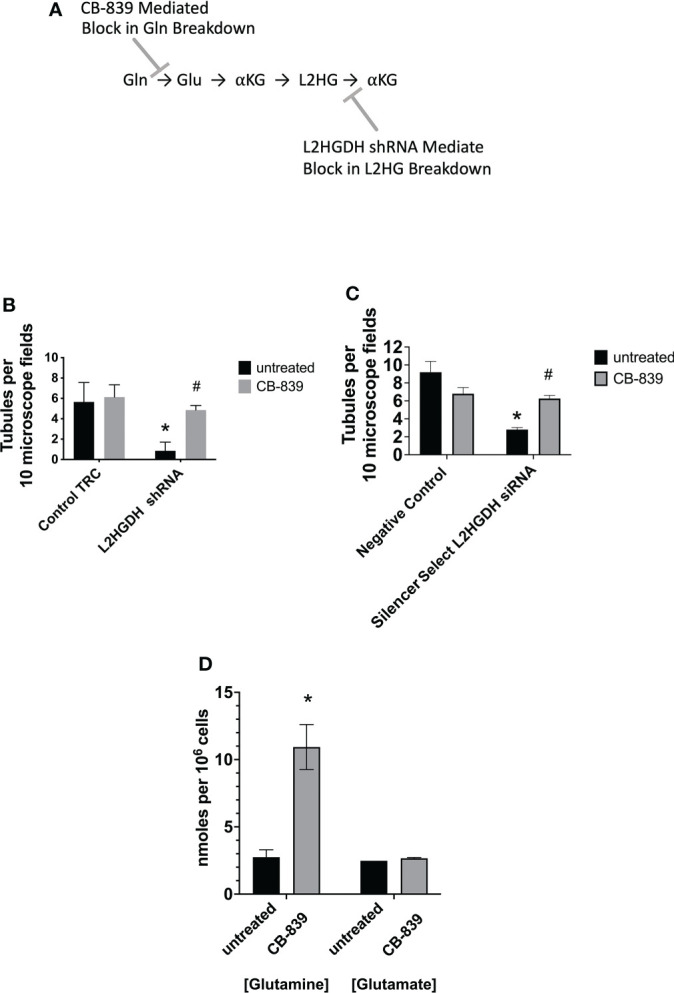
Effect of Glutaminase Inhibitor CB-839 on tubulogenesis. **(A)** Model for the Effect of CB-839 on Gln metabolism. Shelar et al. (2) previously presented evidence indicating that L2HG in RCCs primarily originates from Gln, initially occurring *via* the metabolism of Gln to Glu by Glutaminase. L2HG is subsequently metabolized to αKG by L2HGDH. **(B)** Primary RPT cultures were transduced with lentivirus containing either L2HGDH shRNA or Control TRC shRNA, selected with puromycin, and passaged into matrigel, as described in the **Figure 1** legend. **(C)** Primary RPT cell cultures were transfected with Silencer Select L2HGDH siRNA or Negative Control siRNA, and passaged into matrigel 1 day later, as described in the **Figure 1** legend. In parts **(B**, **C)** Matrigel cultures were incubated with DMEM/F12- IT further supplemented with 5 ng/ml EGF and either 1 µM CB-839 or no further supplement, the day after cultures were initiated in matrigel. In parts **(B**, **C)**, tubules were counted as described in the **Figure 1** legend. Values are averages +/- SEM of triplicate determinations. (*) p < 0.05 relative to untreated Control TRC; (#) p < 0.05 relative to the untreated L2HGDH condition. **(D)** The level of glutamine and glutamate was determined in primary RPT cell cultures treated with either 1 µM CB-839 or untreated, as described in Materials and Methods. Values are averages +/- SEM of triplicate determinations. (*) p < 0.05 relative to untreated control in the glutamine condition.

Previously, Shelar et al. (2) conducted studies which indicated that L2HG is primarily generated from Glutamine (Gln) in RCCs. The proposed pathway is illustrated in **Figure 2A**. Consistent with this hypothesis, the glutaminase inhibitor CB-839 was observed to reduce L-2HG levels in ccRCC cells (2). Thus, the effect of the glutaminase inhibitor 1 µM CB-839 on tubulogenesis by primary RPTs was examined. **Figure 2B** shows that incubation of primary RPTs transduced with lentiviral L2HGDH shRNA with 1 µM CB-839 prevented the decrease in the number of tubules caused by L2HGDH shRNA. In contrast, 1 µM CB-839 did not significantly affect the number of tubules in matrigel cultures transduced with the Control TRC vector. Similarly, as shown in **Figure 2C**, CB-839 significantly alleviated the decrease in tubulogenesis caused by Silencer Select L2HGDH siRNA. **Figure 2D** shows that 1 µM CB-839 caused a significant increase in intracellular glutamine levels, indicating that glutaminase was significantly inhibited under these conditions. Thus, these results are consistent with the hypothesis that the decrease in tubule formation normally caused by the L2HGDH shRNA can be attributed to an increase in the L2HG level, and that this increase no longer occurs in the presence of CB-839.

In order to evaluate this hypothesis further, the effect of extracellular L2HG on tubule formation was examined. The intracellular level of L2HG has been reported to vary dramatically in normal cells derived from different tissues, including 0.4 µM L-2HG in macrophages (27) and 43.79 µM in HEK293FT cells, derived from human kidney (28), and 2HG levels high as 700 µM in white blood cells (29). While the level of D- and L-2HG in the renal microenvironment has not been precisely determined, 1.37 µM 2HG has been measured in serum (30).

**Figure 3:**
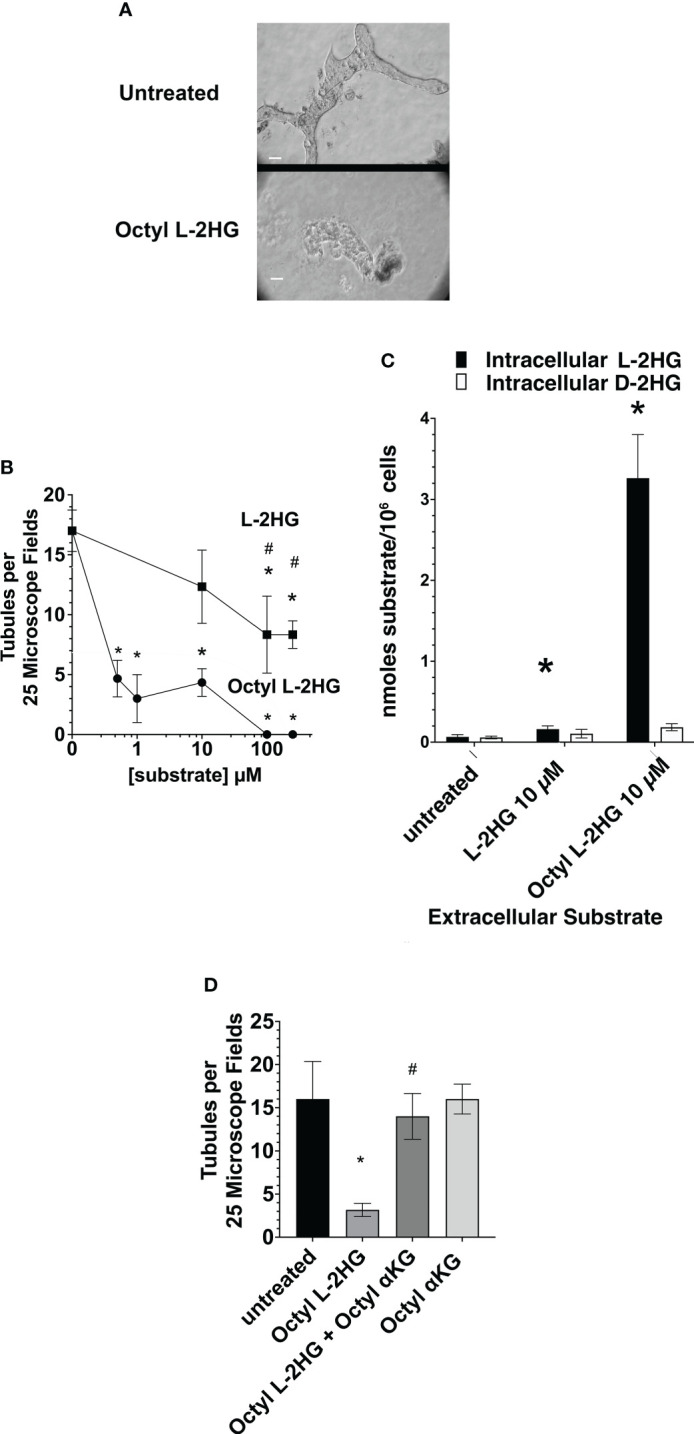
Inhibition of tubulogenesis by Octyl L-2HG. **(A)** Representative microscope fields of either control matrigel cultures in DMEM/F12-IT supplemented with 5 ng/ml EGF alone, or further supplemented with 100 µM Octyl L-2HG. **(B)** The frequency of tubule formation as a function of the concentration of either Octyl L-2HG or L-2HG. **(C)** Effect of 10 µM L-2HG or Octyl L-2HG on the intracellular L- and D-2HG concentration. **(D)** Effect of 5-octyl-αKG (250 µM) on the Octyl L-2HG (100 µM)-induced inhibition of tubulogenesis. Values are averages +/- SEM of triplicate determinations. (*) p < 0.05 relative to untreated cultures; (#) p < 0.05 relative to Octyl L2HG. Scale Bar, 50 µm.

Thus, initially the effect of the cell permeable octyl L-2HG on tubulogenesis was examined. **Figure 3A** shows the typical impairment in tubule formation in matrigel cultures treated with 1 µM octyl L-2HG. As shown in **Figure 3B** as the octyl L-2HG concentration was gradually increased to 100 µM, the number of tubules decreased to 0. In contrast, as the L-2HG concentration was increased to 100 µM, tubule formation only decreased by 50%. **Figure 3C** shows that the 75% decrease in tubule formation observed at 10 µM octyl L-2HG was associated with a forty-fold increase in intracellular L-2HG. Presumably then, the inhibitory effect of octyl L-2HG can be attributed to the increased intracellular L-2HG, which inhibits αKG-dependent dioxygenases. Consistent with this hypothesis, the inhibitory effect of octyl L-2HG on tubule formation was alleviated by 5-octyl-αKetoglutarate (αKG) (as shown in **Figure 3D**). This latter observation can be explained if 5-ocytl-αKG successfully competes with the elevated L-2HG for binding to αKG-dependent dioxygenases, thereby preventing the inhibitory effect of the elevated L-2HG on αKG-dependent dioxygenases.

### 3.2 Effect of L2HGDH KD on L- and D-2HG levels

**Figure 4:**
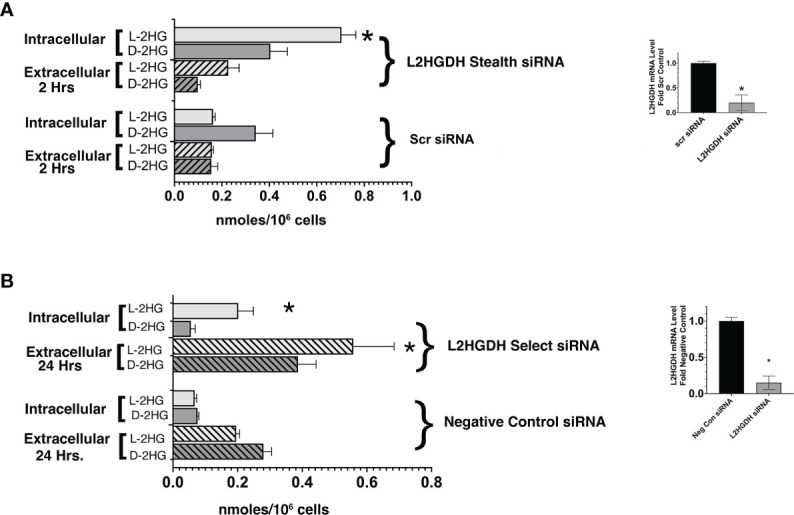
Effect of L2HGDH shRNA an L2HGDH siRNA on the intra- and extracellular L- and D-2Hydroxyglutarate Level. **(A)** Primary RPT cells were transfected with either L2HGDH Stealth siRNA or the corresponding Scrambled siRNA. The medium was changed on Days 1, 2 and 3 post-transcription. **(A)** Two hours after the medium change on Day 3 post-transfection, frozen cell pellets were prepared, and medium was collected. **(B)** Primary cultures were transfected either with L2HGDH Silencer Select siRNA or Negative Control siRNA. As in part A, the medium was changed on Days 1, 2 and 3 post-transfection. The medium that was changed on Day 3, however, was collected and frozen, while frozen pellets were prepared 2 h after the final medium change. The L- and D-2HG in each of the samples (i.e. cell pellets and medium) was derivatized, separated by GC/MS, and quantitated, as described in Materials and Methods. Values are averages (+/- SEM) of triplicate determinations. In part A, *p < 0.05 relative to intracellular L-2HG with Scr siRNA, while in part B, *p < 0.05 relative to Negative Control siRNA in the same condition (i.e. either intracellular L-2HG, or extracellular L-2HG). In the insets, L2HGDH mRNA levels were determined as described in Materials and Methods. (*) p < 0.05 relative to Control condition.

In order to determine a) whether L2HGHD siRNA causes a significant increase in L-2HG, and b) whether the L-enantiomer, rather than the D- enantiomer is affected, the level of both L- and D-2HG was determined in primary cultures treated with either L2HGDH Stealth siRNA or Scrambled control siRNA by means of a GC-MS analysis. **Figure 4A** shows that in control primary cultures treated with Scr siRNA D- was the major enantiomer of 2HG (0.34 +/- 0.07 nmol D-2HG/10^6^ cells vs. 0.16 +/- 0.01 nmol L-2HG/10^6^ cells). In contrast, in primary cultures treated with L2HGDH stealth siRNA, L- was the major enantiomer. This can be attributed to a 4.4-fold increase in the L-enantiomer of 2HG in primary RPT cells treated with L2HGDH stealth siRNA (to 0.70 +/- 0.06 nmol/10^6^ cells), unlike the D-enantiomer, which did not change significantly (0.40 +/- 0.07 nmol/10^6^ cells). **Figure 4B** shows that similarly, L-2HG became the predominant enantiomer in primary RPT cultures treated with L2HGDH silencer select siRNA (vs. Negative Control siRNA). This can be explained by a significant increase in the level of the L- rather than the D- enantiomer of 2HG in primary RPTs treated with L2HGDH silencer select siRNA. These results are consistent with the hypothesis that L2HGDH stealth siRNA caused a specific increase in L2HG due to a reduction in the level of the L2HGDH enzyme without affecting D2HGDH enzyme levels.

### 3.3 Effect of L2HGDH KD on DNA and histone methylation

Previously, a knockdown of L2HGDH was observed not only to cause an increase in L2HG levels, but in addition to cause alterations in the level of DNA hydroxymethylation and histone methylation in renal carcinomas and other cultured renal cells (31). In this respect, an increase in L2HG has similar consequences to those caused by increases in D2HG (and a decrease in D2HGDH) observed in other types of cancers (32). Such changes in DNA and histone methylation have been attributed to the ability of 2HG to inhibit αKG-dependent dioxygenases that control gene expression (32). Included amongst these dioxygenases are TET methylcytosine dioxygenases, as well as Jumonji domain containing histone demethylases (JMDHs), a family of histone demethylases. Thus, the hypothesis was examined that the inhibition of these two families of αKG-dependent dioxygenases resulting from an L2HGDH knockdown could be responsible for the inhibition of tubulogenesis caused by an L2HGDH knockdown in normal renal cells, as well as other related alterations.

**Figure 5:**
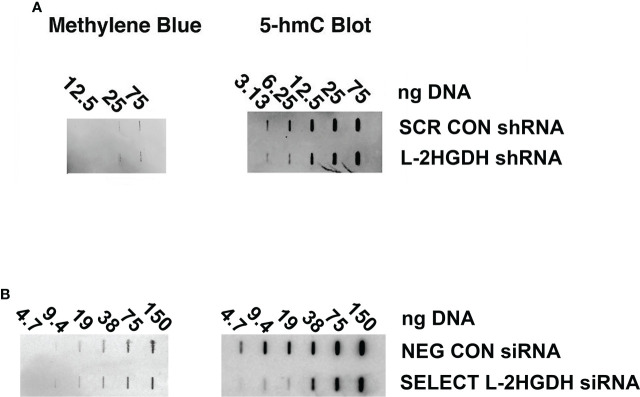
Effect of L2HGHD shRNA and siRNA on DNA methylation. The effect of L 2HGDH shRNA and siRNA on 5hmC levels. Genomic DNA was purified from primary cultures **(A)** transduced with lentivirus containing either Scrambled shRNA or L2HGDH shRNA vectors, or **(B)** transfected with either Negative Control siRNA, or Silencer Select L2HGDH siRNA. Serial dilutions of the genomic DNA were applied to slot blots. Subsequently, blots were probed with a 5hmC antibody, and total DNA visualized with methylene blue.

The TET methylcytosine dioxygenases (including TET1, TET2, and TET2) are involved in the demethylation of 5-methylcytosine (5mC) in genomic DNA (1). The reaction initially involves the formation of 5-hydroxymethyl cytosine (5hmC) from 5mC, to determine whether this enzymatic activity is altered in primary RPT cultures were treated with either L2HGDH shRNA or Scrambled shRNA. Subsequently, genomic DNA was purified from these primary cultures, and the level of 5hmC in the genomic DNA was examined by means of slot blots. **Figure 5A** shows that the level of 5hmC is indeed reduced in genomic DNA derived from cultures treated with L2HGDH shRNA, as compared with Scrambled Controls. Similar results were obtained when the primary cultures were treated with Silencer Select L2HGDH siRNA, as compared with Negative Control-treated cultures (as show in **Figure 5B**). These results are consistent with reduced TET enzymatic activity in primary RPT cell cultures treated with L2HGDH shRNA.

**Figure 6:**
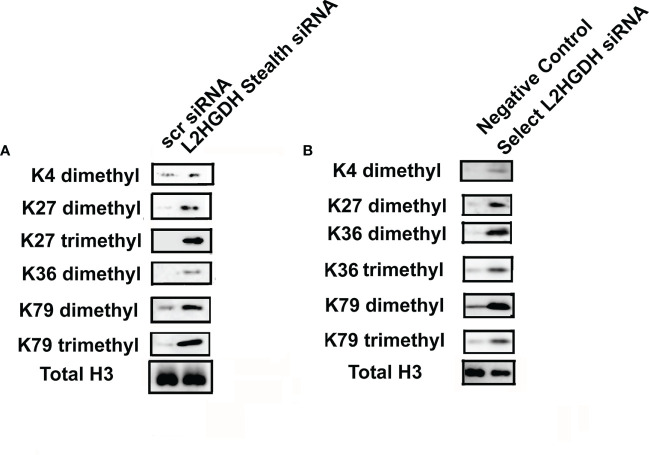
Effect of L2HGDH siRNA on histone methylation. Nuclear histones were purified from primary cultures transfected either with **(A)** L2HGDH Stealth siRNA or Scrambled Control Stealth siRNA, or **(B)** Silencer Select L2HGDH siRNA or Negative Control siRNA. Three days later, nuclear histones were purified. Subsequently, nuclear histones were separated by SDS/PAGE, transferred to nitrocellulose, followed by Western analysis, as described in Materials and Methods.

Inhibition JMDHs would also be expected to occur following an increase in L-2HG levels, which would prevent the demethylation of some classes of histones. Thus, the effect of L2HGDH siRNA on methylated histones was examined. **Figure 6A** shows that there was a generalized increase in the level of methylated histones in primary cultures treated with L2HGH Stealth siRNA, as compared with parallel cultures with Scrambled Control siRNA. Not only did the level of K4 dimethyl histone H3 increase, but in addition, there was an increase in the level of both K27 dimethyl and trimethyl histones in L2HGDH Stealth siRNA treated cultures. The level of K36 dimethyl histone increased in an analogous manner in the L2HGDH Stealth siRNA treated cultures. In addition, K79 dimethyl and trimethyl Histone H3 levels increased in primary RPT cells treated with L2HGDH Stealth siRNA. As shown in **Figure 6B**, similar increases in histone methylation were observed in primary cultures treated with L2HGDH Silencer Select siRNA. Thus, our results are consistent with the hypothesis that both DNA and histone methylation increase in primary RPT cell cultures with an L2HGDH KD.

### 3.4 Effect of an L2HGDH KD on the expression of differentiated transporters

**Figure 7:**
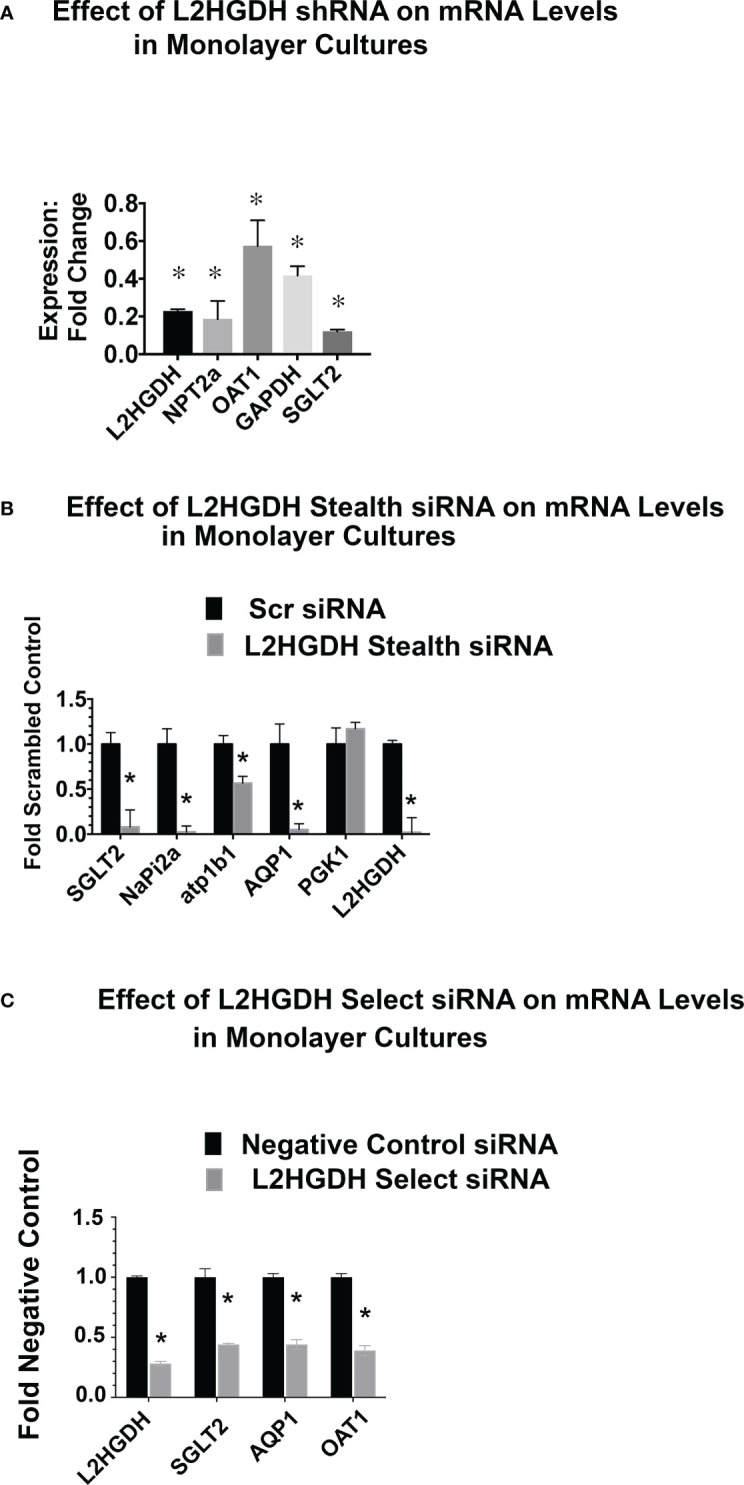
Effect of L2HGDH shRNA and L2HGDH siRNA on the expression of transporter mRNAs in monolayer cultures. **(A)** The expression of the mRNAs for L2HGDH, NPT2a, OAT1, GAPDH and SGLT2 was determined in primary cultures transduced with lentivirus containing either an L2HGDH shRNA or Control TRC vector. The relative expression of mRNAs in cultures transduced with L2HGDH shRNA was compared to the level in cultures transduced with Control TRC shRNA. **(B)** Relative mRNA levels were determined in primary RPT cells transfected twice with either L2HGDH Stealth siRNA or Scrambled siRNA. **(C)** Relative mRNA levels were determined in primary RPT cells transfected twice with either L2HGDH Silencer Select siRNA or Negative Control siRNA, as described in Materials and Methods. In part A, (*) p < 0.05 relative to either the TRC Control (Part A), the Scrambled Control (Part B), or, the Negative Control (Part C).

The expression of RPT transporters is induced during the tubulogenesis which occurs during kidney development (12, 33). Included amongst these transporters are the Na^+^/phosphate cotransporter (NPT2a), the p-Aminohippurate transporter (OAT1), Aquaporin 1 (AQP1), and the Na^+^/glucose cotransporter (SGLT2). Initially, the effect of an L2HGDH KD was examined in monolayer cultures. **Figure 7A**. shows the reduced NPT2a, OAT1 and SGLT2 mRNA levels in monolayer cultures of primary RPT cells transduced with lentiviral L2HGDH shRNA. Transporter mRNA levels were similarly reduced in primary RPT monolayers transduced with L2HGDH Stealth siRNA (vs. Scrambled Controls) (**Figure 7B**), as well as L2HGDH Silencer Select siRNA (**Figure 7C**).

**Figure 8:**
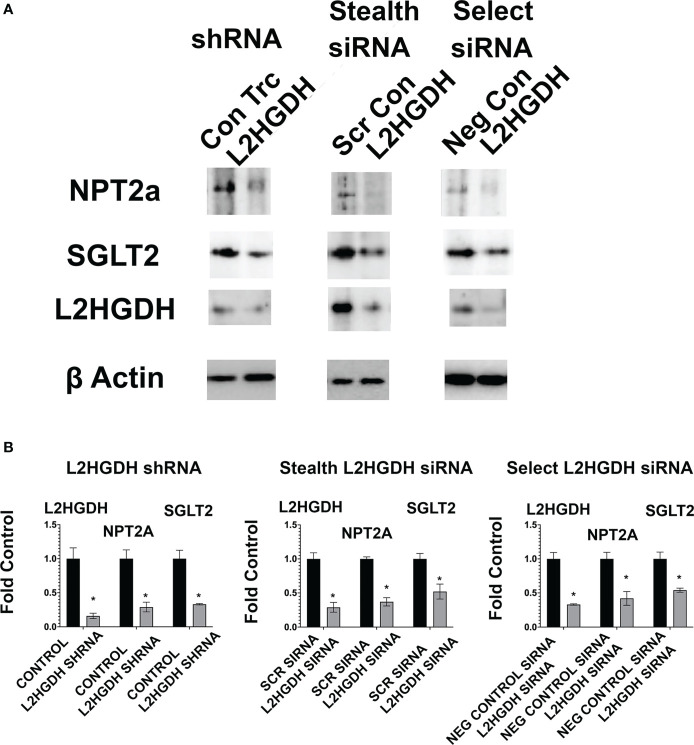
Effect of lentiviral L2HGDH shRNA and L2HGDH siRNA on the level of the NPT2a, SGLT2 and L2HGDH proteins in monolayer cultures. **(A)** Blots of shRNA and siRNA-treated primary cultures. Blots used in the study were prepared following Western transfers of SDS/PAGE gels, as described in Materials and Methods. The samples in the blots were derived from lysates of primary cultures treated either with (i) L2HGDH shRNA (or Control TRC shRNA), (ii) L2HGDH Stealth siRNA (or Scrambled siRNA), or (iii) L2HGDH Silencer Select siRNA (or Negative Control siRNA). **(B)** Relative Levels of NPT2a, SGLT2 and L2HGDH. The relative levels of NPT2a, SGLT2 and L2HGDH were determined using ImageLab software. Values are averages (+/- SEM) from duplicate bands for each sample. (*) p < 0.05 relative to the TRC Control (for L2HGDH shRNA), the Scr Control (for L2HGDH Stealth siRNA), and the Negative Control (for L2HGH Silencer Select siRNA).

The effect of an L2HGDH KD at the protein level was also examined. **Figures 8A** **, B** show that in primary RPTs treated with L2HGDH shRNA, the level of the NPT2a and SGLT2 proteins was reduced by 67 +/- 11% and 58 +/- 11%, respectively, as compared with Con TRC shRNA-treated controls. Similar reductions in the level of NPT2a and SGLT2 proteins were observed in primary cultures treated with either L2HGDH Stealth siRNA or L2HGDH Silencer Select siRNA, as compared with their respective controls. **Figures 8A** **, B** also shows a substantial reduction in the L2HGDH protein level in primary cultures treated with L2HGDH shRNA, L2HGDH Stealth siRNA, or L2HGDH Silencer Select siRNA, as compared with their respective controls (Con TRC shRNA, Scrambled Stealth siRNA, and Negative Control siRNA, respectively).

Relative to their respective controls, the L2HGDH protein levels were 16 +/- 4% (L2HGDH shRNA), 29 +/- 7% (L2HGDH Stealth siRNA), and 33 +/- 1% (L2HGDH Silencer Select siRNA).

### 3.5 Effect of basement membrane on gene expression, as well as on alterations caused by L2HGDH knockdowns

#### 3.5.1 Effect of L2HGDH KD on Transporter gene expression: Influence of matrigel

Previous studies indicate that the basement membrane components of matrigel promote the differentiation of cells originating from a diverse number of tissues (34). Thus, it is reasonable to determine whether the expression of differentiated renal transporters is altered by basement membrane matrigel, as well as by the process of tubulogenesis itself (35). For this reason, the effect of basement membrane matrigel on the expression of transporter mRNAs and the cellular response to L2HGDH shRNA was examined, including studies both in Control RPT cell cultures (treated with lentiviral Con TRC shRNA), as well as in RPT cell cultures treated with lentiviral L2HGDH shRNA.

**Figure 9:**
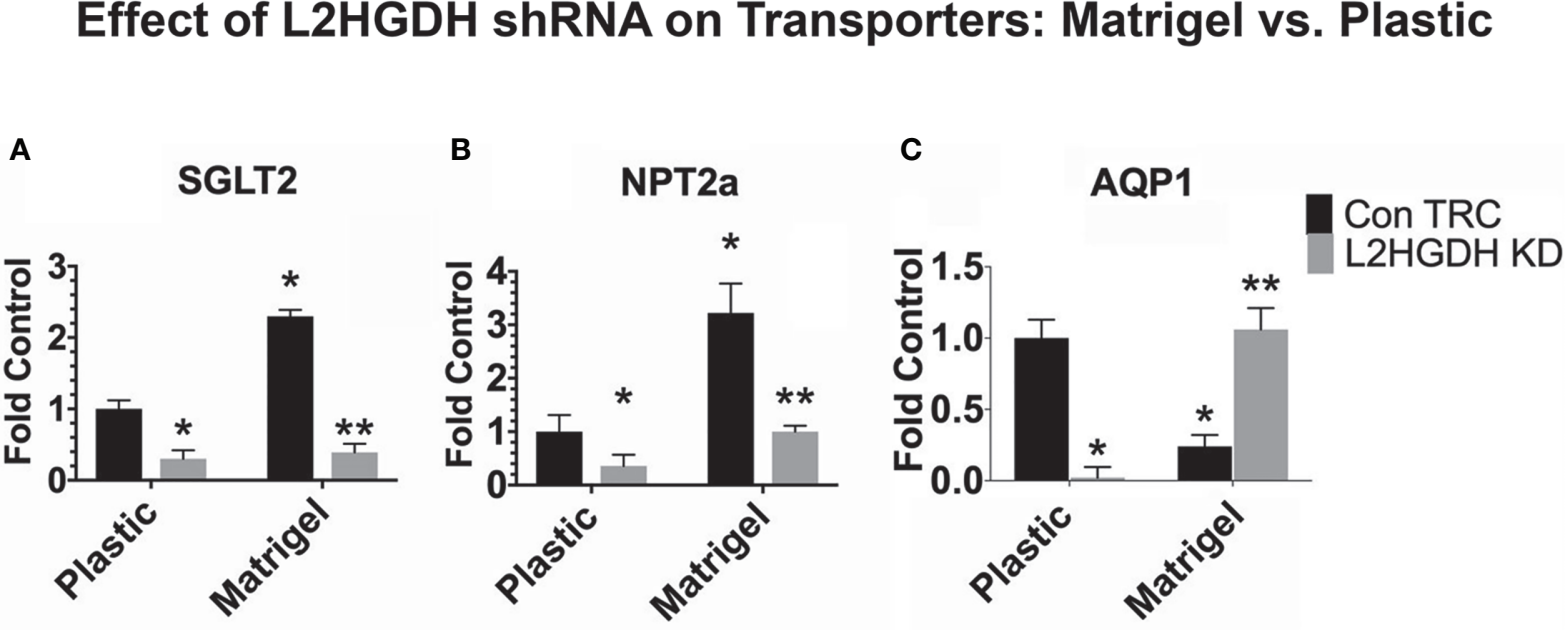
Effect of matrigel on transporter gene expression. Primary cultures of RPT cells were transduced with lentiviral L2HGDH shRNA or Control TRC shRNA. A portion of the cultures was transferred into either matrigel or onto plastic. The relative level of mRNA for **(A)** SGLT2, **(B)** NPT2a, and **(C)** AQP1 was determined as described in Materials and Methods. (*) p < 0.05 for the Con TRC Plastic Control; (**) p < 0.05 for the ConTRC Matrigel Control.

As shown in **Figure 9**, the expression of SGLT2 mRNA and NPT2a mRNA increased when Control primary RPT cell cultures were maintained in matrigel (where they form tubules) as compared to plastic (as shown in **Figures 9A** **, B**, respectively). The expression of SGLT2 and NPT2a mRNA was reduced in matrigel as well as in monolayer cultures transduced with L2HGDH shRNA (**Figures 9A** **, B**). In contrast, **Figure 9C** shows that the AQP1 mRNA level increased in matrigel cultures transduced with L2HGDH shRNA, unlike the Control TRC cultures, which exhibited a decreased level of AQP1 mRNA in matrigel. This unexpected observation with AQP1 mRNA (which distinguishes AQP1 from the two other transporters studied) may possibly be attributed to the role of AQP1 in cell adhesion and migration, in addition to transport (36).

#### 3.5.2 Effect of L2HGDH KD on HNF transcription factor expression: Influence of matrigel

**Figure 10:**
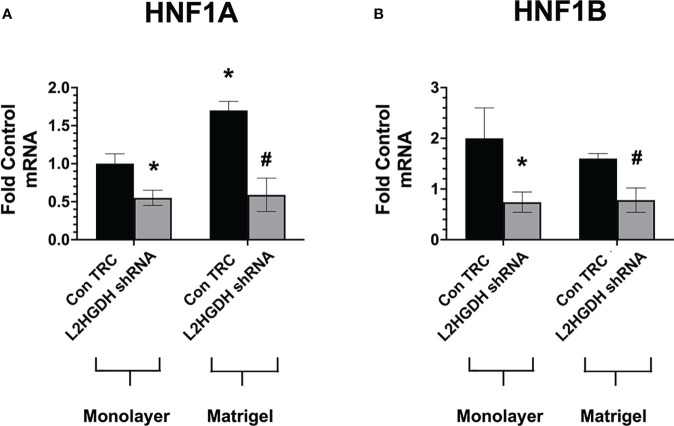
Effect of L2HGDH shRNA on expression of HNF1α and HNF1α. Primary RPT cells were transduced with lentiviral L2HGDH shRNA or Control TRC shRNA. A portion of the cultures was transferred either into matrigel or onto plastic. The relative level of mRNA for **(A)** HNF1α and **(B)** HNF1β was determined as described in Materials and Methods. (*) p < 0.05 relative to Con TRC Monolayers; (#) p < 0.05 relative to Con TRC Matrigel cultures.

Hepatocyte nuclear factors (HNFs) not only regulate the expression of a number of renal transporters (13) but also play a role in kidney development (37). Thus, the decreased expression of renal transporter mRNAs observed in cultures treated with L2HGDH shRNA may possibly be explained by reduced expression of HNFs in RPT cell cultures treated with lentiviral L2HGDH shRNA. To examine this hypothesis, the expression of the mRNAs encoding for 2 HNFs, HNF1α, and HNF1β was examined both in monolayer and matrigel cultures treated with either lentiviral L2HGDH shRNA or Control TRC shRNA. The level of HNF1α and HNF1β mRNA was significantly reduced in matrigel cultures transduced with L2HGDH shRNA (vs. Control TRC cultures), as shown in **Figures 10A** **, B**, respectively. In addition, a significant reduction in the HNF1α and HNF1β mRNA was observed in monolayer cultures transduced with L2HGDH shRNA (**Figure 10A**). Thus, these results are consistent with the hypothesis that a reduction in the level of HNF1α and HNF1β contributes to reduced expression of transporter mRNAs as well as reduced tubulogenesis caused by L2HGDH shRNA.

#### 3.5.3 Effect of L2HGDH KD on other genes affecting tubulogenesis: Influence of matrigel

**Figure 11:**
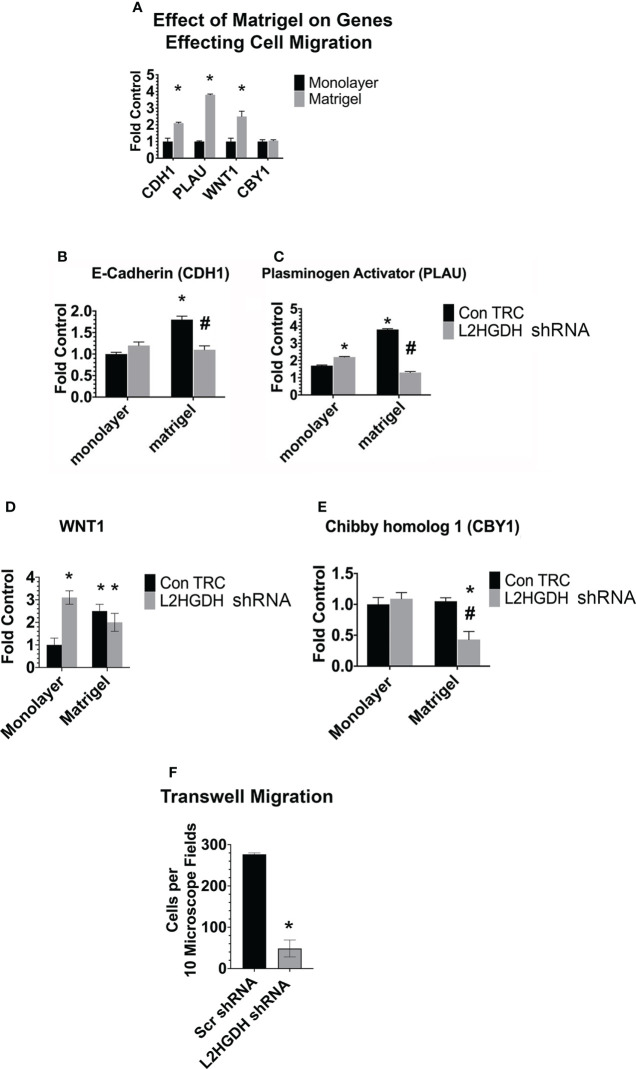
Expression of genes affecting tubulogenesis, and cell migration. **(A)**. The relative level of mRNA encoding for E-Cadherin (CDH1), tissue Plasminogen Activator (PLAU), Wnt1 and Chibby homolog 1 (CBY1) was determined in parallel monolayer and matrigel cultures. The relative level of the mRNA encoding for **(B)** E-Cadherin, **(C)** Plasminogen Activator, **(D)** WNT1, and **(E)** CBY1 (1) was determined in monolayer and matrigel cultures tranduced with lentiviral L2HGDH shRNA or Control TRC shRNA. **(F)** EGF-stimulated cell migration across PET membranes was determined in RPT cells following their transduction with lentiviral L2HGDH shRNA or Con TRC shRNA. (*) p < 0.05 relative to monolayer cultures in the same condition in panel **(A)**; In panels **(B–E)**, (*) p < 0.05 relative to the Con TRC monolayer culture condition; in 11F (*) p < 0.05 relative to the scr shRNA condition; (#) p < 0.05 relative to the Con TRC Matrigel Condition **(E)**.

The expression of other genes which affect tubulogenesis was also examined both in matrigel and monolayer cultures. **Figure 11A** shows that the level of E-Cadherin (CDH1), urokinase type-Plasminogen Activator (PLAU), and Wingless/Integrated 1 (Wnt1) mRNA increased significantly in matrigel, as compared with monolayer cultures, unlike Chibby 1 (CBY1). However, similar increases in the level of CDH1 and PLAU mRNA were not observed in L2HGDH shRNA-derived matrigel cultures (**Figures 11B** **, C**). Indeed, in matrigel cultures treated with L2HGDH shRNA, the PLAU mRNA level declined to a level significantly below that observed in Control monolayer cultures. In contrast, Wnt1 mRNA increased substantially in monolayer cultures transduced with lentiviral L2HGDH shRNA, and this increased level of Wnt1 mRNA was maintained in matrigel cultures with an L2HGDH KD (**Figure 11D**). In contrast, the level of CBY1 mRNA decreased in matrigel cultures with an L2HGDH KD (**Figure 11E**). CBY1 is a negative regulator of β-catenin-mediated transcriptional activation (38), and thus a reduction in CBY1 gene expression would be expected to stimulate Wnt signaling *via* β-catenin.

Decreases in the expression of E-cadherin and urokinase type-Plasminogen Activator may very well result in reduced cell migration, and, as a consequence, reduced tubulogenesis in EGF treated matrigel cultures. Thus, the effect of EGF on cell migration through transwells was examined. **Figure 11F** shows that the stimulatory effect of EGF on the migration of RPT cells transduced with lentiviral L2HGDH shRNA was reduced greater than six-fold. This observation is consistent with the hypothesis a reduction in cell migration through matrigel contributes to the reduced tubulogenesis observed in RPT cells transduced with lentiviral L2HGDH shRNA.

## 4 Discussion

Previously, the studies of Shelar et al. (2) indicated that “the L2HG/L2HGDH axis” plays a significant role in the development of RCCs. The expression of a number of genes which possess high-CpG-density promoters was altered because of the elevated L2HG levels in RCCs, including Polycomb proteins, which target developmentally regulated genes (2). The increased L2HG in RCCs may very well contribute to the altered signal transduction pathways observed in these tumors, including signaling pathways involving EGF and Wnt (39–41), as well as PI3K/Akt/mTOR (42, 43) and VHL/HIF (44). Both the EGF and Wnt mediated signaling pathways control kidney development and differentiation (45, 46). Thus, changes in these signaling pathways would be expected to alter the differentiated state, and to select for stemness. Indeed, recent studies indicate that these pathways are activated in renal cancer stem cells (41, 47).

In this report, evidence is presented that EGF-induced renal proximal tubulogenesis is controlled by the L2HG/L2HGDH axis. As we have previously reported, tubulogenesis by primary RPT cells in matrigel normally occurs in response to EGF (9). However, here we present evidence indicating that tubulogenesis in matrigel is inhibited when L2HGDH is knocked down by lentiviral L2HGDH shRNA. Consistent with the hypothesis that L2HG is involved in mediating the inhibitory effect of EGF on tubulogenesis, a) the L2HG level increased in cultures with an L2HGDH knockdown, b) L2HG octyl-ester inhibited tubulogenesis, and c) the glutaminase inhibitor CB-839 prevented the inhibitory effect of L2HGDH shRNA on tubulogenesis. This latter observation can be explained if CB-839 causes a decline in L2HG levels, similar to that reported by Shelar et al. (2) in RCC cells treated with CB-839. This latter observation can be explained as being the consequence of the inhibition of the glutamine (Gln) metabolic pathway leading to L2HG (the pathway being Gln → Glutamic (Glu)→ αKetoglutarate (αKG)→ L2HG) (2).

Our metabolomic studies indicate that an L2HGDH KD results in an increase in L2HG levels7, which is presumably responsible for the inhibition of tubulogenesis. Both L- and D-2HG are competitive inhibitors of α-KG dependent dioxygenases. A considerable number of α-KG dependent dioxygenases are present in mammalian cells, including enzymes affecting metabolic processes, in addition to enzymes affecting the methylation of DNA and histones.

Our recent metabolomic studies (unpublished) also indicate that the level of a number of metabolites whose synthesis depends upon α-KG dependent dioxygenases is indeed altered in primary RPT cells treated with L2HGDH siRNA. For example, the level of saccharopine is reduced in cultures treated with L-2HGDH siRNA. Saccharopine is produced from lysine and α-KG by the lysine-ketoglutarate reductase domain of α-aminoadipate semialdehyde synthase (48). Similarly, the increased level of leucine can be explained by reduced leucine metabolism to 4-methyl-2-oxopentanoate by L-leucine-2-oxoglutarate aminotransferase (α-KG receiving the NH2-group from leucine) (49). The contribution of these metabolic changes to the altered tubulogenesis observed in these studies is unclear.

However, it is likely that the L2HG-mediated changes in differentiation observed in these studies can be attributed to the inhibition of those DNA and histone demethylases which are included amongst the family of αKG-dependent dioxygenases. Included amongst the DNA demethylases which are αKG-dependent dioxygenases, and inhibited by 2HG, are TET1, TET2, and TET3 enzymes. The TETs sequentially remove the methyl group from 5methylcytosine, by a series of successive oxidations which include the initial formation of 5hmC. Our 5hmC slot blotting study indicates that the level of 5hmC increased in primary RPT cells treated with L2HGDH siRNA, consistent with the inhibition of TETs by L2HG in this condition. Thus, the inhibition of tubulogenesis by L2HG can possibly be explained by the inhibition of DNA demethylation. Consistent with this hypothesis, αKG octyl ester alleviated the inhibition of tubulogenesis caused by L2HG-octyl ester, presumably by preventing competitive inhibition of TET enzymes by L2HG.

Our experimental results show a generalized increase in the level of methylated histones in primary RPT cell cultures treated with L2HGDH siRNA. A similar observation was made in 3T3-L1 cells with a 2HG-producing IDH2 mutation (5). In the 3T3-L1 cells expressing this IDH2 mutation, the increase in histone methylation (as well as DNA methylation) was associated with a block which prevented their differentiation into adipocytes (5). The increased histone methylation observed in these 3T3-L1 cells was attributed to the inhibition of JHDMs by 2HG (5, 50). Consistent with this hypothesis, Lu et al. (5) observed that differentiation of 3T3L1 cells into adipocytes was similarly impaired when the histone demethylase KDM4C was inhibited. KDM4C is a H3K9me3 demethylating JHDM. These latter studies suggest that the block in tubulogenesis observed in our RPT cultures transduced with lentiviral L2HGDH shRNA is also a consequence of repressive H3K9 trimethylation. Consistent with this hypothesis, H3K9me3 is downregulated in nascent nephrons during kidney development (51).

The effect of an L2HGDH knockdown on the expression of mRNAs encoding for RPT transporters was also examined in our studies. The level of Npt2a and Sglt2 mRNA was substantially reduced in matrigel cultures which had been transduced with lentiviral L2HGDH shRNA (as observed in monolayer cultures). These observations can be explained if the expression of the genes encoding for Npt2a and Sglt2 (SLC34A1 and SLC5a2, respectively) is repressed due to increased methylation of CpG islands present within their promoters and/or the promoters of HNF transcription factors (presumably due to the inhibition of TET enzymes). Consistent with the hypothesis of gene repression due to promoter methylation is the observation that the SLC5a2 gene is present within a differentially methylated region (DMR) of genomic DNA, that is hypomethylated in the RPT, unlike other tissues (52).

Consistent with the hypothesis that HNF transcription factors are involved are studies indicating that the expression of SLC34A1 and SLC5a2 is dependent upon the binding of HNF1α and HNF4α to their promoters (13). Similarly, the expression of renal OATs depends upon the binding of HNF1α and HNF1β to the promoter region, which in turn is controlled by the DNA methylation status (13, 53). The expression of the genes encoding for the HNF family of transcription factors themselves can also be suppressed by methylation of their promoters. For example, the methylation of 4 CpG sites in the HNF1A promoter results in the silencing of the HNF1A gene, and its downstream targets, such as GnT-4a glycosyltransferase in pancreatic β cells (54). The HNF4A gene is similarly silenced by DNA promoter methylation (5mC), as exemplified by liver progenitors, whose differentiation depends upon TET mediated formation of 5hmC, resulting in the expression of HNF4A, and the initiation of a hepatocyte developmental program (55).

Although DNA 5mC hypermethylation is a characteristic of cells that overproduce 2HG, increased histone methylation is also observed in cells which overproduce 2HG. Indeed, Schvartzman et al. (4) and Lu et al. (5) have proposed that the block in adipocyte differentiation and myocyte differentiation caused by 2HG is a consequence of increased histone H3K9 methylation rather than a rise in DNA methylation. However, in nephron progenitors, the Polycomb proteins EZH1 and EZH2 maintain stemness by stimulating H3K27 trimethylation. EZH2 reportedly suppresses expression of HNF1β (56), as well as HNF1α (57), presumably by stimulating H3K27 trimethylation. The increased level of H3K27me3 in primary RPTs treated with L2HGDH siRNA may similarly be responsible for the inhibition of tubulogenesis in matrigel (58). However, we cannot rule out the involvement of other trimethylated histones, and/or methylated CpG islands, given that similar results were obtained with the EHMT1/2 inhibitor Unc0638 and the DNA methylase inhibitor 5AzaC (unpublished).

A consequence of increased DNA and histone methylation may very well include reduced expression of transcription factors such as HNF1α and HNF1β. Indeed, epigenetic silencing of both the HNF1A and HNF1B genes has been reported and has been attributed to the methylation of CpG islands in the promoters of these genes (54, 59). Thus, reduced expression of HNF1α, and HNF1β may contribute to the inhibition of tubulogenesis caused by L2HGDH shRNA. Both HNF1α and HNF1β play significant, but distinct roles in kidney development (37, 60, 61). HNF1β appears when the nephrogenic mesenchyme is induced to form a polarized epithelium (which involves Wnt signaling) (37, 62, 63). HNF1β is also involved in segmentation of the developing nephrons, which involves Notch signaling (64). HNF1α appears after HNF1β, playing a role in formation of renal proximal tubules, including the expression of differentiated RPT transporters (e.g., Npt2a, SGLT2 and OAT1) (13, 65).

In our experimental studies, we examined the effects of an L2HGDH KD on the expression of a number of mRNAs and their respective proteins, expressed in the RPT, unlike other nephron segments. Consistent with our observations, are the results of high-throughput technologies employed to quantitatively analyze the transcriptomes, proteomes, and genomes of mammalian cells (66). A central conclusion that has come from this work is that protein levels at steady state are primarily determined by mRNA levels (66). Admittedly, this relationship between protein and mRNA levels is not necessarily maintained during “dynamic” adaptation processes, and during short-term temporal adaptions post-transcriptional processes are important (66). However, our studies with primary RPT cells treated with lentiviral shRNA were conducted more than 10 days after lentiviral transduction. Although the effects of an L2HGDH knockdown on mRNA levels were also examined following transfection with L2HGDH siRNA, the results obtained were very similar to those obtained with an L2HGDH KD obtained with lentiviral L2HGDH shRNA. A very significant aspect of our studies was the observation that the expression of a number of the mRNAs encoding for differentiated RPT transporters was higher in matrigel cultures, as opposed to monolayer cultures. However, the mRNA levels were examined after 1 week in matrigel, and under these conditions, a reduction in the level of SGLT2 and NPT2a mRNAs was still observed, similar to results observed in parallel cultures maintained on a plastic substratum.

Recently, cancer stem cells (CSCs) with activated Wnt and Notch signaling, have been isolated from clear cell RCCs (41). The activation of Wnt signaling (observed in RPT monolayers with an L2HGH KD) occurs when the level of the Wnt antagonist DKK1 declines, an event which may result from the hypermethylation of the DKK1 promoter, the trimethylation of H3K27, and the recruitment of the Polycomb complex, as observed in lung cancers (67). In contrast, the activation of Notch signaling may occur when STRAP (serine-threonine kinase associated protein) interacts with the Polycomb complex, so as to inhibit H3K27 methylation, which as a consequence increases the expression of the Notch effectors HES1 and HES5, as observed in colorectal CSCs (68). Unlike the case with Wnt and Notch signaling, HNF1α- and HNF4α-mediated signaling is reduced in RCCs (69, 70), resulting in reduced expression of distinctive RPT genes, such as SLC34A1 (NaPi2a) and SLC22A6 (OAT1) (71). In contrast, the expression of AQP1 often increases in RCCs (72, 73), similar to the increased expression of AQP1 mRNA in RPT matrigel cultures with an L2HGDH KD.

To summarize, this report has evaluated the effects of an L2HGDH KD on the differentiation of normal RPT cells, the cell of origin of ccRCCs. Evidence is presented that EGF-induced tubulogenesis is inhibited by L2HG itself, as well as an L2HGH KD, which frequently occurs in ccRCCs. We have conducted metabolomic studies which indicate that the L2HGDH KD results in a significant increase in L-2-hydroxygutarate. The inhibition of tubulogenesis caused by an L2HGDH KD was associated with reduced expression of a number of mRNAs encoding for differentiated transporters expressed in the RPT, as well as reduced expression of mRNAs encoding for transcription factors which regulate the expression of these transporter mRNAs. The reduced expression of these mRNAs can be attributed to the increased DNA and histone methylation which occurred as a consequence of an L2HGDH KD. In addition, our studies indicate that EGF-induced cell migration was impaired as a consequence of an L2HGDH KD, which could be explained by reduced expression of mRNAs encoding for such proteins as plasminogen activator. Thus, the reduction in tubulogenesis observed in normal cells with elevated 2HG can be attributed to the impairment of functions required for the process of tubulogenesis, as well as dedifferentiation.

## Data availability statement

The raw data supporting the conclusions of this article will be made available by the authors, without undue reservation.

## Ethics statement

The animal study was reviewed and approved by Institutional Animal Care and Use Committee of the University at Buffalo.

## Author contributions

MT has directed and conducted studies with primary cultures of renal proximal tubule cells both as monolayer and matrigel cultures, in addition to preparing the manuscript. NM conducted studies which led to the measurement of L- and D-2Hydroxyglutarate, in addition to interpreting the results. JT directed the GC/Mass Spec studies, assessing the results and making appropriate scientific changes that led to the final definitive results. SS determined the direction of the overall studies, and made arrangements for appropriate experiments to be conducted in cases where the experimental direction required the involvement of others, such as JT and NM. All authors contributed to the article and approved the submitted version.

## Funding

The funding received for the research in the laboratories of SS and MT was obtained from the NCI RO1CA200653. The funding for the research in the laboratory of JT was obtained from GM grant R35GM119557. Funds for open access publication fees are from the University at Buffalo Foundation.

## Acknowledgments

The RealTime PCR and cell migration data in this study was acquired at the Optical Imaging and Analysis Facility, School of Dental Medicine, State University of New York at Buffalo, in addition to The Confocal Microscopy and Flow Cytometry Center of the Jacob School of Medicine and Biomedical Sciences of The State University of New York at Buffalo. In addition, we thank the members of the Indiana University Mass Spectroscopy Facility for assistance in optimizing the GC/Mass Spectrometry protocol. The research reported in this article was supported in part by RO1CA200653 to Sunil Sudarshan. JT is supported by the National Institute of General Medical Sciences of the National Institutes of Health under Award Number R35GM119557.

## Conflict of interest

The authors declare that the research was conducted in the absence of any commercial or financial relationships that could be construed as a potential conflict of interest.

## Publisher’s note

All claims expressed in this article are solely those of the authors and do not necessarily represent those of their affiliated organizations, or those of the publisher, the editors and the reviewers. Any product that may be evaluated in this article, or claim that may be made by its manufacturer, is not guaranteed or endorsed by the publisher.
